# Overnight 1-mg DST Serum Cortisol in Various Stages of Chronic Kidney Disease—Normative Data and Underlying Mechanisms

**DOI:** 10.1210/jendso/bvae002

**Published:** 2024-01-12

**Authors:** Robin Garg, Saba Samad Memon, Anurag Lila, Vijaya Sarathi, Priyanka Patil, Tukaram Jamale, Sreyashi Bose, Manjiri Karlekar, Virendra Patil, Nalini Shah, Tushar Bandgar

**Affiliations:** Department of Endocrinology, Seth GS Medical College and KEM Hospital, Mumbai, 400012, Maharashtra, India; Department of Endocrinology, Seth GS Medical College and KEM Hospital, Mumbai, 400012, Maharashtra, India; Department of Endocrinology, Seth GS Medical College and KEM Hospital, Mumbai, 400012, Maharashtra, India; Department of Endocrinology, Vydehi Institute of Medical Sciences and Research Center, Bangalore, 560066, Karnataka, India; Department of Endocrinology, Seth GS Medical College and KEM Hospital, Mumbai, 400012, Maharashtra, India; Nephrology, Seth GS Medical College and KEM Hospital, Mumbai, 400012, Maharashtra, India; Nephrology, Seth GS Medical College and KEM Hospital, Mumbai, 400012, Maharashtra, India; Department of Endocrinology, Seth GS Medical College and KEM Hospital, Mumbai, 400012, Maharashtra, India; Department of Endocrinology, Seth GS Medical College and KEM Hospital, Mumbai, 400012, Maharashtra, India; Department of Endocrinology, Seth GS Medical College and KEM Hospital, Mumbai, 400012, Maharashtra, India; Department of Endocrinology, Seth GS Medical College and KEM Hospital, Mumbai, 400012, Maharashtra, India

**Keywords:** ONDST, chronic kidney disease, dexamethasone suppressed cortisol, Cushing, CKD, ONDST cortisol

## Abstract

**Context:**

Data on the overnight 1 mg-dexamethasone suppression test (ONDST) in renal dysfunction are limited.

**Objective:**

We aim to determine the normative range of ONDST cortisol across chronic kidney disease (CKD) stages and reasons for its alteration.

**Methods:**

Prospectively, 180 CKD (30 each in G2-G5/5D) patients and 30 healthy controls underwent ONDST 8 Am serum cortisol (chemiluminescent immunoassay [CLIA]). In an exploratory cohort, 45 (15 each: G3b/G4, G5/G5D, and healthy controls) individuals’ blood biochemistry for basal (8 Am) cortisol and adrenocorticotropin (ACTH), post-ONDST 8 Am dexamethasone, ACTH, cortisol (CLIA and liquid chromatography–tandem mass spectrometry), and 4 Pm cortisol was collected.

**Results:**

Post-ONDST cortisol (µg/dL) correlated inversely (*r* = 0.47; *P* < .005) with estimated glomerular filtration rate (eGFR) (mL/min/1.73 m^2^), with 95th percentile being 1.2 in controls, 3.0 in G2, 3.2 in G3a, 4.3 in G3b, 4.7 in G4, 5.7 in G5, and 7.1 in G5D. In the exploratory cohort, basal 8 Am cortisol and ACTH, and post-ONDST dexamethasone were similar among controls and CKD subgroups. ONDST ACTH (for evaluating the hypothalamo-pituitary-adrenal axis) was slightly higher in G5/5D vs controls (8.9 vs 6.1 pg/mL), while it was similar in G3b/G4 vs controls. Median 8 Am ONDST cortisol was similar on CLIA and LC-MS/MS in controls and higher on CLIA in G3b/4 (1.7 vs 1.1 µg/dL; *P* = .012) and G5/5D (2.4 vs 1.7 µg/dL; *P* = .002) than LC-MS/MS. Post-ONDST serum cortisol drop from 8 Am to 4 Pm was significant in controls (0.5-<0.2 µg/dL) and G3b/4 (1.7-1.2 µg/dL), but not in G5/5D (2.4-2.2 µg/dL).

**Conclusion:**

The normative data of ONDST serum cortisol with eGFR-based cutoffs are useful in evaluating Cushing syndrome in CKD. Prolonged cortisol half-life and immunoassay-related assay cross-reaction are likely contributors to higher ONDST cortisol.

Patients with chronic kidney disease (CKD) have upregulated hypothalamo-pituitary-adrenal axis (HPA), and it is in a state of nonneoplastic hypercortisolism [1]. Diagnosing Cushing syndrome (CS) in patients with CKD is challenging due to overlapping nonspecific clinical features, altered biochemical tests, and equivocal radiology [2]. Among the available biochemical diagnostic tests, 24-hour urinary free cortisol (24-h UFC) is not recommended in these patients as urinary cortisol excretion decreases markedly below the creatinine clearance of 60 mL/minute [3, 4]. Even in patients with diagnosed Cushing disease, 24-h UFC was normal or decreased in the presence of renal dysfunction [2, 5]. Therefore, the Endocrine Society guidelines (2008) suggest that a normal (low) midnight serum or salivary cortisol value or a normal response to 1-mg dexamethasone probably excludes endogenous CS in CKD [3]. At the time of guideline formulation, the diagnostic thresholds in patients with CKD for midnight serum cortisol, late-night salivary cortisol (LNSC), or overnight 1-mg dexamethasone suppression test (ONDST) serum cortisol were not well established; hence, given the low quality of evidence, the suggestion was primarily based on expert opinion. Subsequently, one study reported normative ranges of LNSC in CKD stages 1 to 4 (milder degrees of CKD, n = 20 in each stage) and another in CKD stage 5D (end-stage renal disease [ESRD] on dialysis, n = 16) [6, 7]. Hence, the latest update of the guidelines suggests LNSC as a preferred test in CKD, although CKD stage-specific cutoffs for diagnosis are not well established [8].

Historically (in the 1980s), few studies reported that patients with ESRD have poor suppressibility to ONDST [9-13]. Various factors like HPA axis upregulation, altered dexamethasone or cortisol metabolism, and assay cross-reactivity have been proposed mechanisms for the same [9-13]. However, studies with a holistic evaluation of the contribution of these factors to poor cortisol suppressibility to ONDST in CKD are limited [14, 15]. Although LNSC has been the most preferred first-line test to evaluate CS in CKD recently, more than one screening tests are often essential to rule in or rule out endogenous CS. Therefore, establishing reference ranges for ONDST cortisol across CKD stages may enhance this test's utility in evaluating CS in CKD. Studies primarily focusing on the normative data of ONDST serum cortisol for various CKD stages are limited [6, 16]. Thus, we aim to establish a normative range of ONDST serum cortisol across all CKD stages (study cohort). We further aim to study the plausible mechanisms involved in the altered suppressibility of cortisol in the CKD cohort by recruiting an additional exploratory cohort.

## Materials and Methods

This prospective study was conducted at the Seth GS Medical College and KEM Hospital, a tertiary care hospital in western India, after approval from the institutional ethics committee and registration in the Clinical Trials Registry of India (CTRI/2019/09/021393). Two cohorts were included—a study cohort (to study the normative range of ONDST) and an exploratory cohort (to study the possible mechanisms involved—dexamethasone levels, and adrenocorticotropin [ACTH] after ONDST). A total of 180 patients with CKD being followed up at the nephrology outpatient department and 30 healthy controls with an estimated glomerular filtration rate (eGFR) greater than or equal to 90 mL/min/1.73 m^2^ were included in the study cohort. Patients with CKD were classified as per the Kidney Disease Improving Global Outcomes (KDIGO) criteria, and 30 patients in each subgroup—G2 (60-89 mL/min/1.73 m^2^), G3a (45-59 mL/min/1.73 m^2^), G3b (30-44 mL/min/1.73m^2^), G4 (15-29 mL/min/1.73 m^2^), G5 (<15 mL/min/1.73 m^2^, not on maintenance dialysis), and G5D (on maintenance dialysis)—were recruited [17]. Patients or controls were excluded if they had acute illness/active infection, acute kidney injury, were pregnant or breastfeeding, or were on drugs that could interfere with cortisol-binding globulin (CBG) levels or dexamethasone metabolism. After written informed consent, baseline characteristics like age, sex, height, weight, and serum creatinine were recorded, and eGFR was calculated using the Chronic Kidney Disease Epidemiology Collaboration (CKD-EPI) equation [18]. All included participants were asked to take a 1-mg dexamethasone tablet orally at 11 Pm, and a blood sample for serum cortisol measurement by chemiluminescent immunoassay (CLIA) was collected the next day at 8 Am.

In an additional exploratory cohort (n = 45), 15 individuals were recruited in the following subgroups: healthy controls (eGFR ≥90 mL/min/1.73 m^2^), CKD G3b/G4, and CKD G5/G5D. Their baseline demographic data were recorded, and a blood sample was collected at 8 Am for serum cortisol (by CLIA) and plasma ACTH. They were asked to take a 1-mg dexamethasone tablet orally at 11 Pm, and blood samples were collected the following day at 8 Am for serum cortisol (by both CLIA and liquid chromatography-tandem mass spectrometry [LC-MS/MS]), dexamethasone, and plasma ACTH. These tests were performed to understand dexamethasone bioavailability and HPA axis suppressibility. Cortisol was measured by both CLIA and LC-MS/MS to rule out cross-reactivity to altered cortisol metabolites in CKD by immunoassay. The dexamethasone bioavailability was considered good if 8 Am serum dexamethasone levels were greater than 1.0 ng/mL [19]. An additional blood sample for serum cortisol (by CLIA) was collected at 4 Pm the same day to understand cortisol metabolism.

The cortisol values were assessed by a solid-phase competitive CLIA method (Advia Centaur CP, Siemens Healthcare) with intra-assay and interassay coefficients of variation being 6.3% and 7.3%, respectively, and an analytical sensitivity of 0.2 µg/dL. The measurement of serum cortisol by the LC-MS/MS method was as described in our previous study [20]. Plasma ACTH was measured by a solid-phase, 2-site sequential CLIA (Liaison, Diasorin) with 4.9% and 8.9% intra-assay and interassay coefficients of variation and analytical sensitivity of 1.6 pg/mL. Serum dexamethasone levels were also measured by LC-MS/MS with a lower limit of quantification of 0.05 ng/mL and an upper limit of 50.05 ng/mL. To convert values of ACTH in pg/mL to pmol/liter, multiply by 0.2202; and cortisol in μg/dL to nmol/liter, multiply by 27.59.

### Statistical Analysis

Statistical analysis was performed using SPSS software version 25.0 (IBM). Categorical data were expressed as absolute numbers and percentages and continuous data as median and interquartile range (first and third quartile). The Mann-Whitney *U* test or one-way analysis of variance was used to compare continuous variables between groups. The Pearson correlation test was used to estimate correlations between eGFR and ONDST cortisol, and logarithmic regression analysis was used to describe the relationship between the two. A 2-sided *P* value less than .05 was considered statistically significant.

## Results

### Study Cohort

**Figure 1.**
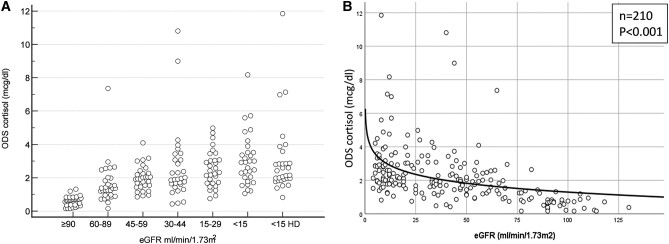
A, The distribution of 8 Am serum cortisol by immunoassay after 1-mg overnight dexamethasone suppression test across varied eGFR categories and B, correlation of ODS cortisol with eGFR. eGFR, estimated glomerular filtration rate; ODS, overnight dexamethasone suppression test.

The baseline characteristics of 180 patients with varying degrees of renal insufficiency (6 subgroups) and 30 controls are described in Table 1. Overall, the male-to-female distribution was 147:63 and the median body mass index (BMI) was 23.8 (21.2-27.0); these were similar among the subgroups and controls. The median age of controls (27 years) and patients with CKD G5D (36 years) were lower than patients across other CKD subgroups (51-61 years). Per the analysis of variance test, ONDST serum cortisol (on CLIA) levels significantly differed across the groups (Fig. 1A). Median ONDST serum cortisol in controls (0.7 µg/dL) was significantly lower than the G3a (1.9 µg/dL), G3b (2.0 µg/dL), and G4 (2.4 µg/dL) CKD subgroups. Further, ONDST serum cortisol in G5 (2.9 µg/dL) and G5D (2.3 µg/dL) CKD were significantly higher than in controls and all other CKD subcategories. The ONDST serum cortisol correlated inversely with eGFR (*r* = 0.47; *P* < .005) with regression equation y = -0.763ln(x) + 4.806, where y: ODST serum cortisol (µg/dL), intercept: 4.806, slope: 0.763 and x: eGFR (mL/min/1.73 m^2^) (Fig. 1B).

**Table 1.** Baseline characteristics and normative range of overnight dexamethasone suppression test 8 Am serum cortisol in study cohort

	eGFR subcategories, mL/min/1.73m^2^						
	≥90 n = 30	60-89 n = 30	45-59 n = 30	30-44 n = 30	15-29 n = 30	<15 n = 30	<15^a^ n = 30
Age, y	27 (23-31)	60 (54-63)	62 (51-65)	61 (50-70)	54 (45-62)	51 (42-62)	36 (28-51)
Male:Female	17:13	23:7	25:5	26:4	17:13	17:13	22:8
Body mass index	22.5 (20.2- 25.9)	26.3 (22.7-27.6)	23.4 (22.6-27.3)	25.4 (22.3-28.1)	25.9 (23.2-29.5)	22.4 (18.7-24.9)	21.1 (18.7-23.2)
Serum creatinine, mg/dL	0.93 (0.84-1.06)	1.09 (0.98-1.20)	1.44 (1.33-1.54)	1.85 (1.65-2.03)	2.77 (2.45-3.34)	5.08 (4.54-6.44)	7.61 (6.00-9.29)
eGFR, mL/min/1.73m^2^	97 (90-106)	69 (65-78)	50 (47-54)	36 (34-41)	21 (19-25)	11 (9-12)	8 (6-9)
Serum 8 Am cortisol post ONDST, µg/dL	0.7 (0.4-0.8)	1.4 (0.9-2.0)	1.9 (1.5-2.3)	2.0 (1.6-3.2)	2.4 (1.7-3.2)	2.9 (2.0-3.6)	2.3 (1. 9-3.1)
95th percentile cutoff for serum 8 Am cortisol post ONDST, µg/dL	1.2	3.0	3.2	4.3	4.7	5.7	7.1

Data are represented as median (interquartile range Q_1_-Q_3_).

To convert values of ACTH in pg/mL to pmol/liter, multiply by 0.2202; and cortisol in μg/dL to nmol/liter, multiply by 27.59.

Abbreviations: ACTH, adrenocorticotropin; eGFR, estimated glomerular filtration rate; ONDST, 1-mg overnight dexamethasone suppression test.

^*a*^On hemodialysis.

The normative ranges (5th-95th percentile) of ONDST serum cortisol across eGFR categories were 0.2 to 1.2 µg/dL in healthy controls, 0.5 to 3.0 µg/dL in G2, 0.9 to 3.2 µg/dL in G3a, 0.5 to 4.3 µg/dL in G3b, 0.9 to 4.7 µg/dL in G4, 1.2 to 5.7 µg/dL in G5, and 1.4 to 7.1 µg/dL in G5D. The 95th percentile of ONDST serum cortisol across eGFR categories was 1.2 µg/dL in healthy controls, 3.1 µg/dL in eGFR 45 to 89 mL/min/1.73 m^2^, and 6.4 µg/dL in eGFR less than 45 mL/min/1.73 m^2^. Among patients with eGFR less than 45 mL/min/1.73 m^2^, the median ONDST serum cortisol was similar in those with BMI less than 25 compared to those with BMI greater than or equal to 25 (2.4 vs 2.3 µg/dL; *P* = .409). Further, among CKD patients with eGFR greater than or equal to 45 mL/min/1.73 m^2^, the median ONDST serum cortisol was 1.3 vs 0.7 µg/dL (*P* = .599) in patients with BMI less than 25 vs greater than or equal to 25.

### Exploratory Cohort

**Figure 2.**
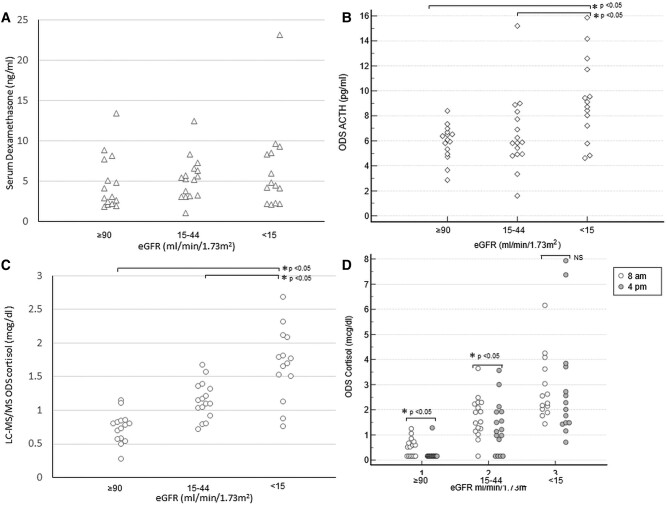
Hormonal biochemistry after 1-mg oral dexamethasone in the exploratory cohort across eGFR category. A, Serum dexamethasone levels (triangles) measured by LCMS/MS; B, ACTH levels at 8 Am (diamond) post ODS; C, ODS serum cortisol (circles) by immunoassay; D, ODS serum cortisol by immunoassay at 8 Am (unfilled circles) and 4 Pm (filled circles). eGFR, estimated glomerular filtration rate; LCMS/MS, liquid chromatography–tandem mass spectrometry; NS, not significant; ODS, 1-mg overnight dexamethasone suppression.

The baseline characteristics of 30 patients with renal dysfunction (subgroups G3b/G4 and G5/G5D) and 15 controls are described in Table 2. Overall, the male-to-female ratio was 32:13, and the median BMI was 23.4 (21.3-26.3) and were similar across eGFR categories. Controls were younger (29 vs 59 years) than patients with eGFR less than 45 mL/min/1.73 m^2^. Basal 8 Am serum cortisol and ACTH were similar among controls and CKD subgroups. ONDST 8 Am serum dexamethasone levels (on LCMS/MS assay) were 4.48 (2.35-8.06), 5.64 (3.77-7.12), and 7.11 (3.76-9.37) ng/mL in controls, G3b/G4, and G5/G5D subcohorts respectively; these differences were statistically insignificant and all individual values were above 1.0 ng/mL (Fig. 2). Post-ONDST ACTH (for evaluating HPA axis) was similar in G3b/G4 vs controls (5.9 vs 6.1 pg/mL; *P* = .83), whereas it was slightly higher in G5/5D vs controls (8.9 vs 6.1 pg/mL; *P* = .03) and G5/5D vs G3b/G4 (8.9 vs 5.9 pg/mL; *P* = .025). Among patients with G5/5D, median 8 Am ONDST cortisol (2.2 vs 2.6 µg/dL on CLIA; *P* = .128 and 1.8 vs 1.7 µg/dL on LC-MS/MS; *P* = .535) was similar in those with post-ONDST ACTH less than 9 pg/mL as compared to post-ONDST ACTH greater than 9 pg/mL. Median 8 Am ONDST cortisol was similar on CLIA and LC-MS/MS in controls (0.5 vs 0.8 µg/dL; *P* = .061) while it was significantly higher on CLIA in G3b/4 (1.7 vs 1.1 µg/dL; *P* = .012) and G5/5D (2.4 vs 1.7 µg/dL; *P* = .002) as compared to LC-MS/MS. The drop in post-ONDST serum cortisol (to estimate cortisol half-life) from 8 Am to 4 Pm was significant in controls (0.5-<0.2 µg/dL; *P* = .011) and G3b/4 (1.7-1.2 µg/dL; *P* = .009), but not in G5/5D (2.4-2.2 µg/dL; *P* = .397).

**Table 2.** Baseline characteristics plus basal (8 Am) and post 1-mg overnight dexamethasone suppression test biochemical parameters at varied times, in exploratory cohort

	eGFR subcategories, mL/min/1.73m^2^		
	≥ 90 (n = 15)	15-44 (n = 16)	<15 (n = 14)
Age, y	29 (23-32)	61 (55-66)	53 (42-60)
Male:Female	9:6	12:4	11:3
Body mass index	21.9 (20.9-23.7)	24.6 (21.7-26.4)	24.2 (21.3-27.6)
Serum creatinine, mg/dL	0.93 (0.70-1.00)	2.20 (1.92-2.55)	5.46 (4.46-8.23)
eGFR, mL/min/1.73 m^2^	105 (93-120)	30 (24-35)	10 (7-14)
Basal values			
8 Am serum cortisol, µg/dL	10.9 (6.2-16.3)	13.7 (9.8-17.1)	13.9 (12.5-16.0)
8 Am plasma ACTH, pg/mL	42.3 (28.5-47.5)	45.5 (23.5-53.1)	43.1 (24.9-54.2)
Post 1-mg oral dexamethasone			
Serum dexamethasone, ng/mL, LC-MS/MS	4.48 (2.35-8.06) (1.88-13.45)	5.64 (3.77-7.12) (1.06-12.44)	7.11 (3.76-9.37) (2.17-23.17)
8 Am plasma ACTH, pg/mL	6.1 (5.0-6.7) (2.9-8.4)	5.9 (4.9-8.3)*^b^* (1.6-15.2)	8.9 (6.9-12.0)*^c^* (4.6-15.9)
8 Am serum cortisol, µg/dL, LC-MS/MS	0.8 (0.6-0.8)*^a^* (0.5-1.8)	1.1 (0.9-1.4)*^b^* (0.7-1.7)	1.7 (1.4-2.1)*^c^* (0.8-2.7)
8 Am serum cortisol, µg/dL	0.5 (0.2-0.7)*^a^* (0.2-1.3)	1.7 (1.2-2.3)*^b^* (0.2-3.6)	2.4 (2.0-3.7)*^c^* (1.4-6.2)
4 Pm serum cortisol, µg/dL	0.2 (0.2-0.2)*^a^* (0.2-1.3)	1.2 (0.3-1.9)*^b^* (0.2-3.6)	2.2 (1.5-3.8)*^c^* (0.7-7.9)

Data are represented as median (interquartile range Q_1_-Q_3_) (range).

To convert values of ACTH in pg/mL to pmol/liter, multiply by 0.2202; and cortisol in μg/dL to nmol/liter, multiply by 27.59.

Abbreviations: ACTH, adrenocorticotropin; eGFR, estimated glomerular filtration rate; LC-MS/MS, liquid chromatography–tandem mass spectrometry.

^*a*^ *P* value less than .05 between eGFR greater than or equal to 90 and eGFR 15 to 44 mL/min/1.73 m^2^.

^*b*^ *P* value less than .05 between eGFR 15 and 44 and eGFR less than 15 mL/min/1.73 m^2^.

^*c*^ *P* value less than .05 between eGFR greater than or equal to 90 and eGFR less than 15 mL/min/1.73 m^2^.

## Discussion

In the present study, we describe the normative range of 1-mg ONDST serum cortisol in CKD patients across all stages. The serum ONDST cortisol was inversely correlated to eGFR, and the 95th percentile of ONDST serum cortisol (µg/dL) was 1.2 in controls, 3.0 in G2, 3.2 in G3a, 4.3 in G3b, 4.7 in G4, 5.7 in G5, and 7.1 in G5D. Declining cortisol suppression to dexamethasone with decreasing eGFR is governed by prolongation of cortisol half-life and assay cross-reaction with cortisol metabolites and is unlikely due to alteration in dexamethasone pharmacokinetics. HPA axis upregulation may be a minor contributor to poor ONDST suppression in ESRD.

The Endocrine Society guidelines suggest using a combination of 24-h UFC, LNSC, and ONDST to screen for CS [3]. However, in patients with CKD, excretion of conjugated cortisol metabolites by the kidney decreases with the worsening of creatinine clearance; hence, 24-h UFC may be falsely negative as a screening test [21]. LNSC is a sensitive test as it measures altered circadian rhythm, the earliest abnormality in CS [7]. In patients with CKD, the circadian rhythm may be altered, and high LNSC values have been reported in CKD stage G5 compared to healthy controls [7]. The study by Cardoso et al [6] evaluated LNSC in the early stages of renal disease and observed an inverse correlation of LNSC with eGFR. Hence, robust CKD stage-specific normative data for LNSC are essential but are unavailable, limiting its utility in CKD.

The available literature on ONDST cortisol in CKD is given in Table 3. In the 1980s, most ESRD studies (n ≤ 10) reported decreased suppression of ONDST cortisol, and they used varied assay methodologies and higher cutoffs ranging from 4 to 5 µg/dL for the definition of suppression [9-11, 13]. Data on ONDST serum cortisol in the early stages of renal disease is limited to 2 studies [6, 16]. Oguz et al (2003) [16] were primarily looking at midnight-to-morning urinary cortisol increment in patients with CKD, describing values of mean ONDST serum cortisol in G2 (n = 5), G3 (n = 12), G4 (n = 13), and G5 (n = 16) CKD stages, and found no difference among these subgroups and controls (n = 14). This data interpretation must be read with caution as the controls had mean ONDST serum cortisol values as high as 2.3 ± 0.86 µg/dL. Cardoso et al (2016) [6] do not mention the actual values of ONDST serum cortisol in the early stages of renal disease (G2-G4) and describe the data as suppressors vs nonsuppressors. Our study is a single-center, prospective study with the primary aim of establishing the range of ONDST serum cortisol in CKD patients. The large sample size (n = 180) adequately represents all CKD stages (6 subgroups) as per eGFR. The data of CKD stage-specific normative range and upper limits of ODST serum cortisol obtained from this study can be applied to reduce false-positive ONDSTs when performed for diagnosis of CS in patients in this cohort. We found an inverse correlation of ONDST serum cortisol to eGFR, reiterating that the use of standardized diagnostic criteria for normal populations should not be applied to CKD patients.

**Table 3.** Studies (with more than 5 patients) reporting dexamethasone suppression test in chronic kidney disease

Year (reference)	No. of patients	CKD stage	Cortisol assay	Dexamethasone measured, yes/no	Cortisol post dexamethasone, µg/dL	Proportion of patients who suppressed	ONDST cortisol cutoff for suppression, µg/dL	ULN ONDST cortisol, µg/dL—95th percentile
1979 [10]	7	G5D	RIA	No	23.1 ± 7.1	None	4.0	Not given
1980 [11]	10	G5D	RIA	No	11.7 ± 7.3	1/10	5.0	Not given
1982 [13]	10	G5D	RIA	Yes	—	None	5.0	Not given
1986 [9]	7	G5D	RIA	Yes	8.7 ± 6.1	None	4.0	Not given
2003 [16]	14	Controls	CLIA	No	2.3 ± 0.9		5.0	Not given
	5	G2			2.3 ± 0.7	All		
	12	G3			2.2 ± 0.7			
	13	G4			2.3 ± 0.6			
	16	G5			2.5 ± 0.5			
2016 [6]	20	G2	RIA	Yes	—	18/20	1.8	Not given
	20	G3			—	17/20		
	20	G4			—	17/20		
2023	30	Controls	CLIA		0.6 ± 0.3	30/30	1.8	1.2
Our study	30	G2			1.6 ± 1.2	21/30		3.0
	30	G3A			2.0 ± 0.7	13/30		3.2
	30	G3B			2.6 ± 2.2	12/30		4.3
	30	G4			2.6 ± 1.1	9/30		4.7
	30	G5			3.1 ± 1.6	7/30		5.7
	30	G5D			3.0 ± 2.2	5/30		7.1
	15	G3B/4	CLIA	Yes	1.7 ± 0.8	8/15		
			LC-MS/MS		1.2 ± 0.3			
	15	G5/5D	CLIA		2.9 ± 1.3	2/15		
			LC-MS/MS		1.7 ± 0.5			

CKD stage is per Kidney Disease Improving Global Outcomes guidelines: G2 60 to 89 mL/min/1.73 m^2^; G3 30 to 59 mL/min/1.73 m^2^; G4 15 to 29 mL/min/1.73 m^2^; G5 less than 15 mL/min/1.73 m^2^; G5D less than 15 mL/min/1.73 m^2^ on dialysis.

To convert values of ACTH in pg/mL to pmol/liter, multiply by 0.2202; and cortisol in μg/dL to nmol/liter, multiply by 27.59.

Abbreviations: ACTH, adrenocorticotropin; CKD, chronic kidney disease; CLIA, chemiluminescent immunoassay; LC-MS/MS, liquid chromatography–tandem mass spectrometry; ONDST, overnight dexamethasone suppression test; RIA, radioimmunoassay; ULN, upper limit of normal.

The ONDST cortisol was higher than the usual cutoff of 1.8 µg/dL in more severe degrees of renal dysfunction. Hence, to evaluate the plausible mechanisms for declining cortisol suppression to dexamethasone with decreasing eGFR, we compared healthy controls and patients with eGFR less than 45 mL/min/1.73 m^2^ in the exploratory cohort.

Altered dexamethasone pharmacokinetics has been one of the plausible mechanisms for decreased cortisol suppressibility to ODST in patients with CKD. A few studies have historically reported impaired dexamethasone oral availability or accelerated metabolism in patients with CKD [12, 13, 22]. However, later studies have shown no significant alteration in dexamethasone pharmacokinetics [9]. The recent study by Cardoso et al (2016) [6] reported similar post-ONDST morning dexamethasone levels across G1 to G4 CKD stages, comparable to the general population. Similarly, in our exploratory cohort, 8 Am serum dexamethasone levels were similar in controls and patients with CKD stages G3/4 and G5/5D. Considering the overall evidence, dexamethasone metabolism is an unlikely reason for the poor cortisol suppressibility in most patients with CKD. Serum dexamethasone levels during the ONDST have been reported in healthy volunteers (Ueland et al 2.8 [1.2-7.3] ng/mL, Ceccato et al 4.9 [3.1-6.3] ng/mL, Genere et al 3.4 [2.5-4.4] ng/mL, Farinelli et al 3.69 [1.4-7.9] ng/mL) with normal renal function, which were similar to our study (4.48 [2.35-8.06] ng/mL) [19, 23-25].

The circadian rhythm is generally preserved in CKD, but nadir plasma ACTH levels were elevated in ESRD and are explained based on subtle glucocorticoid resistance secondary to the inflammatory milieu [7]. Historically, elevated post-ONDST plasma ACTH (on RIA) in ESRD patients has been reported in 2 studies (n ≤ 10) [10, 13]. The role of interfering substances in the serum of patients with CKD was cited as a cause in the first study and lower dexamethasone oral bioavailability in the other. In our exploratory cohort, most patients achieved adequate post-ONDST 8 Am serum dexamethasone levels (≥1 ng/mL) and had suppression of 8 Am plasma ACTH less than 15 pg/mL. ACTH values less than 5 pg/mL were observed in 11 of 45 (24.4%), and between 5 to less than 10 pg/mL in an additional 29 of 45 (64.5%). The clinical significance of the statistically different magnitude of post-ONDST 8 Am plasma ACTH elevation (8.9 pg/mL vs 6.0 pg/mL in ESRD vs normal) is unclear. However, comparable ONDST cortisol between ESRD patients with ACTH below and 9 pg/mL or greater suggests upregulation of the HPA axis as a minor contributor to the poor cortisol suppressibility to ONDST in patients with CKD.

The liver metabolizes cortisol by conversion to the various tetrahydro derivatives followed by conjugation into water-soluble glucuronides, which are then excreted by the kidney. As the renal function declines, conjugated metabolites accumulate and can cause errors in interpreting cortisol values [26]. Nolan et al [26] evaluated several different assays and methodologies in CKD and suggested cross-reaction of metabolites as a reason for varied cortisol levels with different methods. In the exploratory cohort, baseline 8 Am serum cortisol (on CLIA) was similar in normal individuals and patients with CKD G3b/4 and G5/5D. This suggests that interference due to major reduced cortisol metabolites with immunoassay is unlikely or nondiscernable at basal 8 Am levels. On CLIA, the post-ONDST cortisol values were higher than the LC-MS/MS assay in CKD G3b/4 and G5/5D, while this difference was not significant in controls. Thus, immunoassay-related interference due to cortisol metabolites possibly contributes to poor cortisol suppression to ONDST in CKD patients. The interference observed at low values obtained following ONDST demonstrates that the cross-reaction with immunoassay is more prominent at the assay's lower limit of detection.

The prolonged half-life of cortisol in CKD has been reported by numerous studies using a variety of analytical methods [22, 27]. The activity of 11β hydroxysteroid dehydrogenase type 2, which inactivates cortisol (F) to cortisone (E), is probably reduced in patients with ESRD as the mean F/E ratio in ESRD patients was 9.3 vs 4.5 in controls as depicted in a study by N’Gankam et al [28]. Workman et al [9] first proposed that delayed cortisol metabolism in ESRD could be the major contributor to relatively higher post-ONDST serum cortisol levels. Our exploratory cohort data support this hypothesis as with adequate post-ONDST dexamethasone levels and 8 Am plasma ACTH suppression, further decline of ONDST cortisol from 8 Am at 4 Pm was significantly less in the G5/5D CKD stage as compared to the G3/4 CKD stage and controls. The latter may have contribution from the described phenomenon of early escape from dexamethasone suppression or cross-reactivity of the immunoassay with the accumulated cortisol metabolites with declining eGFR [12, 26]. An additional analysis of plasma ACTH and LC-MS/MS serum cortisol at 4 Pm post ONDST could have provided better insights.

The main limitations of our study were that we performed LC-MS/MS ONDST cortisol and dexamethasone levels only in the exploratory cohort, not the entire initial cohort. Nonetheless, we provide a normative range of ONDST serum cortisol across varied degrees of renal dysfunction using commonly used immunoassays that would be widely applicable in routine clinical practice. Further, serum free cortisol levels and CBG were not measured. The alterations in CBG can lead to difficulties interpreting total serum cortisol. Nonetheless, none of our patients had anasarca or low albumin levels, and hence, low CBG may be less likely in our cohort, as reported in a previous study [6]. Milder degrees of cortisol excess in CS can overlap with normal values in CKD, and caution should be exercised in interpretation. The unequal sex distribution between the groups is another limitation of this study. However, as eGFR calculation already incorporates sex and no sex-specific cutoffs for men and women for ONDST cortisol are described in the normal population, it may not alter our study results. This study exclusively includes Asian Indians; however, we believe that the study observations could be generalized to all ethnic groups as no interethnic differences in ONDST cortisol levels have been reported [29]. Nevertheless, further studies are warranted to explore the behavior of ONDST cortisol in CKD patients of various ethnicities.

To conclude, ONDST serum cortisol is inversely correlated with eGFR. The normative data and ONDST serum cortisol cutoffs based on eGFR will be useful in evaluating CS in patients with CKD. Prolongation of cortisol half-life and assay cross-reaction with cortisol metabolites are more likely contributors to declining ONDST cortisol suppression in CKD rather than altered dexamethasone pharmacokinetics or upregulated HPA axis.

## Data Availability

Original data generated and analyzed during this study are included in this published article or in the data repositories listed in “References.”

## Clinical Trial Information

Clinical Trials Registry of India (CTRI/2019/09/021393) (registered September 24, 2019).

## Abbreviations

ACTH: adrenocorticotropin
BMI: body mass index
CBG: cortisol-binding globulin
CKD: chronic kidney disease
CLIA: chemiluminescent immunoassay
CS: Cushing syndrome
eGFR: estimated glomerular filtration rate
ESRD: end-stage renal disease
HPA: hypothalamo-pituitary-adrenal axis
LC-MS/MS: liquid chromatography–tandem mass spectrometry
LNSC: late-night salivary cortisol
ONDST: overnight dexamethasone suppression test
RIA: radioimmunoassay
UFC: urinary free cortisol
